# Effects of Isometric Quadriceps Muscle Exercise with Visual and Auditory Feedback at 1 Year after Total Knee Arthroplasty

**DOI:** 10.1298/ptr.E10260

**Published:** 2024-01-17

**Authors:** Yasutaka KONDO, Yoshihiro YOSHIDA, Takashi IIOKA, Hideki KATAOKA, Junya SAKAMOTO, Yuichiro HONDA, Atsushi NAWATA, Minoru OKITA

**Affiliations:** ^1^Department of Rehabilitation, Japanese Red Cross Nagasaki Genbaku Hospital, Japan; ^2^Department of Orthopedic Surgery, Japanese Red Cross Nagasaki Genbaku Hospital, Japan; ^3^Department of Rehabilitation, Nagasaki Memorial Hospital, Japan; ^4^Department of Physical Therapy Sciences, Nagasaki University Graduate School of Biomedical Sciences, Japan; ^5^Institute of Biomedical Sciences (Health Sciences), Nagasaki University, Japan; ^6^Medical Engineering Laboratory, ALCARE Co, Ltd, Japan

**Keywords:** Total knee arthroplasty, Postoperative pain, Visual and auditory feedback, Physical activity

## Abstract

Objective: To examine the effect of isometric quadriceps exercises with visual and auditory feedback after total knee arthroplasty (TKA). Methods: The sample included 41 patients from our previous study who could be followed up for 1 year after TKA. Patients in the intervention group performed isometric quadriceps exercises with visual and auditory feedback using the quadriceps training machine from the 2nd to the 14th day after TKA, whereas those in the control group underwent standard postoperative rehabilitation (without visual or auditory feedback during isometric quadriceps exercises) in the hospital. Patients were evaluated for pain intensity, timed up and go test (TUG) score, 10-m gait speed, 6-minute walking distance (6MWD), and the Western Ontario and McMaster University Osteoarthritis Index (WOMAC) score 1 year after TKA. Additionally, exercise habits and responses to the International Physical Activity Questionnaire (IPAQ) were investigated. Results: Pain intensity was significantly lower in the intervention group than in the control group. Greater improvements in the TUG test scores, 10-m gait speed, 6MWD, and WOMAC scores were observed in the intervention group. Walking activity, as recorded by the IPAQ, and the proportion of patients with exercise habits were significantly higher in the intervention group than in the control group. Conclusions: These results suggest that performing isometric quadriceps exercise with visual and auditory feedback using the quadriceps training machine has good effects, such as pain reduction, physical function improvement, exercise tolerance, and increased physical activity at 1 year after TKA.

## Introduction

Total knee arthroplasty (TKA) can decrease pain and disability in patients with end-stage knee osteoarthritis (OA)^1)^. Studies have reported that most patients are satisfied with postoperative outcomes of pain relief and functional improvement^2,3)^. However, Bourne et al. reported that 19% of 1703 primary TKA patients were dissatisfied with the outcome^2)^. The primary reason for continued dissatisfaction with TKA is the persistent knee pain^4)^. Wylde et al. reported that approximately 20% of patients experience persistent knee pain despite successful surgery, and long-lasting pain after TKA can affect all dimensions of health-related quality of life by causing functional limitations^5)^. Moderate-to-severe acute postsurgical pain is a major risk factor for long-lasting pain following TKA^6)^. Therefore, the appropriate management of acute pain after TKA is important for prevention of persistent pain and improvement of physical and psychological functions and activities of daily living (ADL).

We conducted a randomized controlled trial (RCT) and found that adjunctive therapy using isometric quadriceps exercises (IQE) with visual and auditory feedback using the quadriceps training machine (QTM) provided early knee pain relief leading to better improvements in physical performance and patient reported outcome of physical function in the early stages of postoperative TKA than those achieved with standard rehabilitation alone^7)^. Previous reports show that distraction of attention to pain reduces acute pain perception^8)^ and goal achievement seems to be related to lower arthritis pain^9)^. Thus, IQE using QTM combined with standard rehabilitation is effective for acute pain after TKA; we hypothesized that postoperative outcomes at 1 year after TKA, such as pain intensity, physical and psychological function, exercise tolerance, physical activity (PA), and ADL, may have also improved in the abovementioned feedback exercise group. Therefore, we followed up participants from the same cohort for 1 year to examine the effect of performing IQE with visual and auditory feedback in adjunct to standard rehabilitation during the early stages of postoperative TKA.

## Methods

### Participants

The study participants were patients older than 60 years diagnosed with knee OA who underwent primary unilateral TKA between April 2017 and August 2018 and were followed up for 1 year after surgery. The exclusion criteria were severe gait disturbance because of which the individual could not walk independently with or without a walking aid, other orthopedic conditions in the lower extremities that limited mobility, significant neurological impairment, severe cardiovascular condition, inability to understand the study objectives due to cognitive impairment and mental disease, and disagreement with the study protocol. Exclusion criteria for the follow-up assessment included patients who developed complications after discharge and those who did not undergo a doctor’s examination 1 year after TKA. The same artificial joint model (cruciate-retaining type) was used for all patients. All patients underwent general intraoperative anesthesia and postoperative femoral nerve block. Written informed consent was obtained from all participants. The study was performed in accordance with the Declaration of Helsinki and approved by the Research Ethical Committee of Japanese Red Cross Nagasaki Genbaku Hospital (approval number: 597). Short-term effects data for this cohort have been published previously^7)^.

### Study flow

The baseline data were assessed the day before their surgery in our hospital. After surgery, all patients received a rehabilitation program during a typical hospital stay of 3 weeks. An evaluation was performed 3 weeks after surgery to examine the short-term effects of rehabilitation during hospitalization. To examine the effects at 1 year after surgery, a follow-up evaluation was conducted on the day the participant visited their orthopedic surgeon for re-examination (368.4 ± 25.6 postoperative days).

### Rehabilitation protocol during hospitalization and intervention

**Fig. 1.**
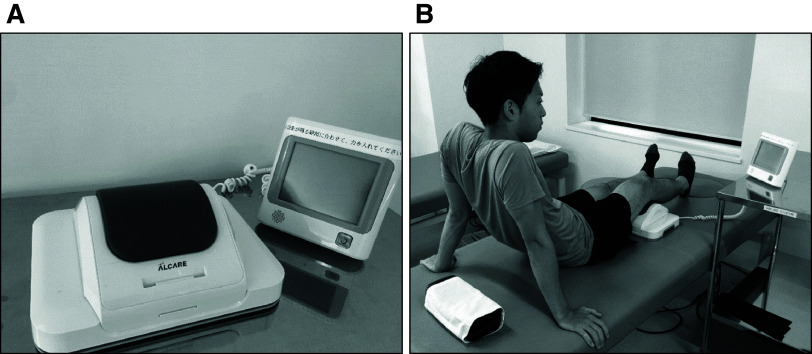
Isometric quadriceps exercise with visual and auditory feedback (A) The QTM (Locomo Scan, Alcare Co., Ltd. Tokyo, Japan). (B) A participant carrying out isometric quadriceps exercise while in the long sitting position by pressing their popliteal fossa on the load measuring part of the QTM. QTM, quadriceps training machine

After surgery, all patients could bear weight on the operated limb, although with varying degrees of tolerance, and the standardized rehabilitation program was started on the first postoperative day. The standardized rehabilitation program consisted of cryotherapy, range of motion (ROM) exercises, and strengthening exercises, including traditional quadriceps sets. Gait training was started on postoperative day 2 and was performed with parallel bars, a walker, a cane, and without aids, depending on the patient’s recovery. In addition, the standardized program included the practice of ADL and stair climbing. Physical therapy sessions were conducted for approximately 1 hour on weekdays for all patients. The control group had the standardized rehabilitation protocol training but received no visual and auditory feedback during IQE. In the intervention group, IQE with visual and auditory feedback were performed instead of the traditional quadriceps sets in the standardized rehabilitation program. The QTM (Locomo Scan; Alcare Co., Ltd., Tokyo, Japan) was developed based on the method for IQE in a long sitting position (Fig. 1). First, the participants measured their maximum isometric quadriceps strength by pressing their popliteal fossa on the load-measuring part of the QTM and then the exercise intensity was determined to 60% of the maximum strength in each training session. The contraction time was 10 seconds, with a 10-second rest between each contraction; participants usually performed two sets of 10 repetitions. During the exercise, the participants kept glancing at the monitor that indicated the value of the pre-set exercise intensity and exerted isometric quadriceps strength to obtain visual feedback. For auditory feedback, music was played from a built-in speaker when the strength reached the determined exercise intensity. This intervention was performed in the intervention group once a day on postoperative days 2 and 3 and twice a day from postoperative days 4 to 14 only during hospitalization. All patients received guidance on home exercises, such as ROM exercises and muscle strength exercises, at discharge.

### Outcome measures

Data were collected prospectively. Data on demographic factors were obtained from electronic medical records. Pain intensity during motion, such as standing up or walking, was determined using a 0–100 mm Visual Analog Scale (VAS). Psychological status was assessed using the validated Hospital Anxiety and Depression Scale (HADS)^10)^ and the Pain Catastrophizing Scale (PCS)^11)^. Isometric knee extension strength of the operated leg was measured using a hand-held dynamometer (μTas F1; ANIMA, Tokyo, Japan) in the seated position. The higher value was normalized to body mass (kg) and used for data analysis. Passive ROM in flexion of the operated knee was measured in the supine position using a standard long-arm goniometer. Physical performance was measured using the timed up and go test (TUG), 10-m gait speed, and 6-minute walking distance (6MWD). The Western Ontario and McMaster Universities Osteoarthritis Index (WOMAC; Japanese version) was used to evaluate self-reported knee joint pain, stiffness, and limitations in physical function^12)^. These were evaluated before, 3 weeks after, and 1 year after TKA. The International Physical Activity Questionnaire (IPAQ; 7-item short version) was investigated only 1 year after TKA. The IPAQ is a self-rating questionnaire used to measure the average amount of PA over a week^13)^. The intensity (walking, moderate intensity, or vigorous intensity), frequency, and duration of PA were recorded, and the min/week for each PA intensity was calculated. In addition, the participants were asked about their exercise habits 1 year after TKA.

### Statistical analysis

All statistical analyses were performed using SPSS version 25.0 (IBM Corp., Armonk, NY, USA). Differences between groups were tested for significance using the unpaired Student’s t-test, with group comparisons for age, height, weight, body mass index (BMI), and PA. Chi-squared tests were used for comparing groups classified as per sex, operated side, presence of OA in contralateral knee, presence of comorbidity, percentage of patients with no pain, and exercise habits. The effects of the intervention on the outcome measures were analyzed using a 3 × 2 (time [before, 3 weeks after, and 1 year after TKA] × group [intervention and control groups]) model in the analysis of variance for split-plot factorial design. Bonferroni post hoc tests were performed for specific comparisons using a two-sided significance level.

## Results

**Fig. 2.**
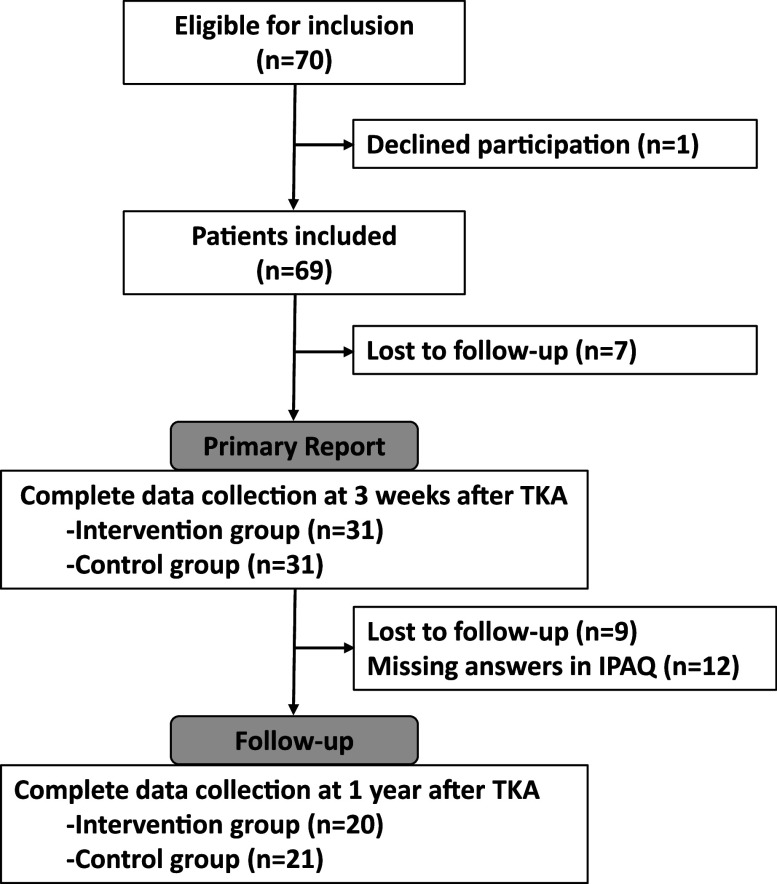
Patient selection flowchart

Figure 2 shows the patient selection process of this study. In this study, we excluded 21 patients because nine were lost to follow-up at 1 year and 12 did not complete the IPAQ. The final study population consisted of 41 participants, with 20 in the intervention group and 21 in the control group.

### Participant characteristics

Only BMI was significantly different in the baseline characteristics of the participants (Table 1). No difference was found between the groups with respect to the dose of postoperative pain medication and distribution of the type of postoperative pain medication in the hospital (p = 0.413 and 0.942, respectively).

**Table 1.** Demographic characteristics of the study participants

Characteristics	Intervention group (n = 20)	Control group (n = 21)	p-value
Age	75.3 ± 5.3	73.1 ± 5.8	0.223
Sex: women, n (%)	16 (80.0)	18 (85.7)	0.471
Height (cm)	152.1 ± 4.7	150.4 ± 6.3	0.356
Weight (kg)	59.3 ± 8.4	64.7 ± 10.8	0.084
BMI	25.7 ± 3.5	28.6 ± 4.3	0.024
Operated knee: right, n (%)	7 (35.0)	10 (47.6)	0.412
OA in contralateral knee, n (%)	13 (65.0)	14 (66.6)	0.910
Grade of OA in operated knee, n			
KL scale 3	0	1	
KL scale 4	20	20	
Grade of OA in contralateral knee, n			
KL scale 2	4	3	
KL scale 3	3	3	
KL scale 4	6	8	
Comorbidity: yes, n (%)	7 (33.3)	6 (30.0)	0.658
Diabetes mellitus	2	2	
Cancer	1	1	
Heart disease	5	3	
Osteoporosis	1	1	

Values are expressed as mean ± SD or n (%).

Statistically significant (p <0.05).

BMI, body mass index; OA, osteoarthritis; KL scale, Kellgren-Lawrence Scale; SD, standard deviation

### Effects of the intervention on outcome measures for both groups

Comparisons of the outcome measures at all time points are presented in Table 2. There were no statistically significant differences in all outcome measures between the two groups before TKA. Three weeks after TKA, the VAS score in the intervention group was significantly lower than that at baseline, and it was also significantly lower than that at 3 weeks after TKA in the control group. At 1 year after TKA, the intervention group showed a significant reduction in VAS scores compared with those at 3 weeks after TKA and also showed better scores than those of the control group (p = 0.003). Furthermore, the proportion of participants who answered “no pain” was significantly larger (p = 0.006) in the intervention group. Similarly, the mean TUG test scores and 10-m gait speed in the intervention group were significantly better than those in the control group (p = 0.020 and 0.010, respectively). Additionally, the mean 6MWD of the intervention group was significantly higher than that of the control group (p = 0.009). The mean WOMAC total score (p = 0.010) and mean WOMAC scores for pain (p = 0.046) and function (p = 0.007) in the intervention group were significantly lower than those in the control group. There was no significant difference in HADS, PCS, isometric knee extension strength, passive ROM in knee flexion, and WOMAC stiffness between the groups 1 year after TKA.

**Table 2.** Group comparisons of outcome measures at all time-points

	Before TKA		3 weeks after TKA		1 year after TKA		*F*-value		
Variable	Intervention group (n = 20)	Control group (n = 21)	Intervention group (n = 20)	Control group (n = 21)	Intervention group (n = 20)	Control group (n = 21)	Time	Group	Group × Time
Pain intensity: VAS score (mm)	53.0 ± 21.0	62.0 ± 23.6	24.3 ± 16.9^a,b^	39.4 ± 22.1^b^	2.8 ± 6.2^a,b,c^	15.9 ± 17.7^b,c^	71.098^e^	11.787^e^	0.299
The number of no-pain (persons)	–	–	–	–	16*	8	–	–	–
HADS									
Anxiety (points)	7.4 ± 4.4	6.9 ± 3.4	3.8 ± 3.1^b^	5.0 ± 4.6	3.9 ± 3.2^b^	4.7 ± 2.6^b^	13.760^e^	0.399	1.039
Depression (points)	5.7 ± 3.3	6.1 ± 3.1	4.1 ± 3.2	5.8 ± 3.8	4.5 ± 3.0	5.6 ± 3.4	2.475	1.388	1.032
PCS total score (points)	29.6 ± 13.6	33.4 ± 12.2	15.7 ± 11.6^b^	20.6 ± 12.6^b^	13.8 ± 10.4^b^	13.3 ± 13.1^b,c^	39.439^e^	0.871	0.924
Knee extension strength (kgf/kg )	0.25 ± 0.11	0.29 ± 0.11	0.19 ± 0.07^b^	0.17 ± 0.07^b^	0.32 ± 0.09^b,c^	0.37 ± 0.11^b,c^	68.021^e^	0.740	3.475^d^
Passive ROM flexion (degrees)	122.5 ± 13.0	118.6 ± 14.5	121.0 ± 6.8	120.5 ± 10.4	116.8 ± 9.2	111.2 ± 13.2^b,c^	10.933^e^	1.188	1.217
TUG (s)	10.5 ± 3.6	12.0 ± 5.5	10.0 ± 2.3	11.9 ± 5.0	7.5 ± 1.5^a,b,c^	9.4 ± 3.3^b,c^	23.869^e^	2.667	0.194
10-m gait speed (m/s)	1.15 ± 0.32	1.07 ± 0.32	1.13 ± 0.25	0.97 ± 0.29	1.55 ± 0.31^a,b,c^	1.32 ± 0.24^b,c^	67.348^e^	3.801	2.295
6MWD (m)	319.2 ± 103.0	282.5 ± 86.0	319.7 ± 83.0	274.3 ± 90.8	423.9 ±79.0^a,b,c^	358.3 ± 74.3^b,c^	44.631^e^	4.328^d^	0.868
WOMAC									
Total (points)	40.6 ± 20.9	38.0 ± 12.2	26.3 ± 12.1^b^	32.2 ± 16.1	11.9 ± 8.0^a,b,c^	20.6 ± 12.1^b,c^	40.907^e^	1.524	2.696
Pain (points)	9.2 ± 4.5	7.7 ± 3.0	5.8 ± 3.3^b^	7.1 ± 3.3	2.1 ± 2.2^a,b,c^	3.9 ± 3.4^b,c^	32.023^e^	0.651	3.304^d^
Stiffness (points)	3.6 ± 2.3	3.1 ± 2.1	2.9 ± 1.5	2.9 ± 1.7	1.3 ± 1.3 ^b,c^	1.8 ± 1.3^b,c^	15.592^e^	0.000	0.914
Function (points)	27.8 ± 15.4	27.1 ± 10.1	17.6 ± 9.2^b^	22.3 ± 12.1	8.5 ± 5.2^a,b,c^	15.0 ± 8.7^b,c^	37.780^e^	1.863	2.107

Values are expressed as mean ± SD. Analyzed using analysis of variance for split-plot factorial design followed by Bonferroni post hoc test.

^a^ Significant group differences (p <0.05). ^b^ Significant differences from before TKA (p <0.05). ^c^ Significant differences from 3 weeks after TKA (p <0.05). ^d^p <0.05; ^e^p <0.01.Analyzed using the Chi-squared test. *Significant vs. Control group (p <0.05).

TKA, total knee arthroplasty; VAS, Visual Analog Scale; HADS, Hospital Anxiety and Depression Scale; PCS, Pain Catastrophizing Scale; ROM, range of motion; TUG, timed up and go test; 6MWD, 6-minute walking distance; WOMAC, Western Ontario and McMaster University Osteoarthritis Index

### Physical activity intensity and exercise habits at 1 year after TKA

Table 3 shows the intensity of PA in both groups 1 year after TKA. The mean walking activity values in the intervention group were significantly higher than those in the control group. However, there was no significant difference in mean values for moderate- and vigorous-intensity activity between the groups. The proportion of patients with exercise habits was significantly higher (p = 0.025) in the intervention group (n = 17, 85.0%) than in the control group (n = 11, 52.4%). “Walking” was the most common type of exercise in both groups. Others were swimming or underwater walking, sports, physical therapy in a clinic, and exercise in a home.

**Table 3.** Physical activity intensity at 1 year after TKA

	Intervention group (n = 20)	Control group (n = 21)	p-value
Walking (METs-min/week)	2,146.7 ± 1381.0	905.9 ± 1182.6	0.004
Moderate (METs-min/week)	312.0 ± 740.1	188.6 ± 732.5	0.595
Vigorous (METs-min/week)	0	22.9 ± 104.7	0.329

Values are expressed as mean ± SD or n (%).

Statistically significant (p <0.05).

TKA, total knee arthroplasty; METs, Metabolic equivalents; SD, standard deviation

## Discussion

In the present study, we included 41 patients from our previous study who were followed up for 1 year after surgery. The participants’ baseline characteristics, except BMI, showed no statistical changes between groups. Previous studies reported that BMI was not identified as a risk factor for persistent pain after TKA^6,14,15)^. Therefore, the difference in BMI between the two groups may have had little effect on the other outcomes.

One year after TKA, the change in VAS scores from preoperative to 1 year after TKA was −50.2 mm in the intervention group and −46.0 mm in the control group, exceeding the minimal clinically important improvement of −19.9 mm for knee OA^16)^. However, the pain intensity (VAS score) was significantly lower in the intervention group than in the control group. In addition, the number of patients who answered “no pain” was significantly higher in the intervention group than in the control group. These results suggest that the intervention prevented persistent postoperative pain, which is a problem with TKA. Our IQE with visual and auditory feedback demands attention to exercise load control and requires the participants to keep glancing at the QTM monitor and listening to the music. Reports show that engaging in tasks that detract attention from pain^8,17)^ and goal achievement can lead to a reduction in pain intensity^9)^. A recent RCT reported that the use of an app-based feedback-controlled active muscle training program can reduce pain in the immediate postoperative period after TKA^18)^. Based on these findings, exercise with attention to visual and auditory stimuli and goal achievement may have helped facilitate significant pain relief up to 3 weeks after surgery, which may have affected decreased pain intensity at 1 year after TKA, resulting in better pain relief.

TUG test score and 10-m gait speed at 1 year after TKA were significantly improved compared with those at 3 weeks after TKA in both groups and the intervention group showed better improvement than the control group did. The improvement in physical performance following TKA is largely attributed to the restoration of muscle strength^19)^, knee joint ROM^20)^, and pain relief^21)^. In this study, there was no significant difference in knee extension strength and passive ROM flexion 1 year after TKA between the groups. In contrast, the intervention group had significantly better pain relief, as indicated by the VAS score, WOMAC pain score, and number of group members who answered “no pain.” Based on these results, better pain improvement, rather than muscle strength and ROM, may have presumably affected the favorable physical performance in the intervention group.

WOMAC a patient-centered self-reported health status questionnaire, its subscale of the physical function has been used to measure patients’ ability to perform certain ADL, including going from sitting to standing, walking, or putting on socks^22)^. Because WOMAC is a patient-reported outcome, a significant decline in WOMAC function scores means a patient’s recognition of improvements in physical function and ADL. It has been reported that ADL is associated with pain^23)^ and physical performance^24)^. Thus, better pain relief and physical performance in the intervention group at 1 year after TKA may lead to an improvement in the ADL that the patient can recognize.

Exercise tolerance measured with the 6MWD test at 1 year after TKA was significantly improved compared to that measured at 3 weeks after TKA in both groups, and the intervention group had a significantly higher 6MWD value than the control group. Moreover, PA intensity evaluated with the IPAQ at 1 year after TKA showed that low-level activity like walking was significantly higher in the intervention group than in the control group. Previous studies demonstrated that subjects with chronic pain had decreased 6MWD compared to control subjects^25)^, and 6MWD was related to PA as measured by the IPAQ^26)^. Meeus et al. observed that exercise tolerance is closely related to pain during activity, particularly if pain is interpreted as harmful^27)^. Although these findings are related to chronic pain, we believe that they can be applied to acute pain, and early acute pain relief may lead to and improve a virtuous cycle of exercise tolerance and PA. Indeed, Chan et al. found that lower pain severity was associated with more time spent walking daily after hospital discharge following TKA^28)^. The lower pain intensity observed at 3 weeks after TKA in the intervention group may have contributed to favorable exercise tolerance capacity and PA at 1 year after TKA. These results show that the intervention group experienced an improvement in PA, followed by an increase in exercise tolerance after TKA and early improvement of pain.

One year after TKA, the intervention group had a higher percentage of exercise habits than the control group did. This result suggests that participants in the intervention group developed a more active lifestyle. A previous study reported that lifestyle and chronic pain are related, with an inactive lifestyle leading to pain^29)^. Thus, an active lifestyle in the intervention group may be the result of early pain relief at 3 weeks after TKA. In addition, an active lifestyle may be related to better results in the intervention group 1 year after TKA.

This study had some limitations. First, since the time point of follow-up was only 1 year after TKA, the recovery process and continuity of exercise after discharge from the hospital were unknown. It would have been better if there had been a follow-up at 3 months or 6 months after surgery. Second, the follow-up rate was rather low, at 64.5% in the intervention group and 67.7% in the control group, which affected the reliability of the study results. Finally, IPAQ and exercise habits were only investigated 1 year after TKA and not preoperatively. At 1 year after TKA, the intervention group had higher PA and rate of exercise habits than the control group, which may have influenced the better results of the intervention group; however, it is also possible that the intervention group had higher PA before TKA. Since there was no difference in preoperative physical performance or function between the two groups, we speculate that the difference in PA was small before TKA.

## Conclusions

Isometric quadriceps muscle exercise with visual and auditory feedback performed in the early postoperative period after TKA reduced acute knee pain intensity better than standard rehabilitation did at 3 weeks after TKA and showed effects in terms of less pain, and better improvement in physical performance, exercise tolerance, and the patient’s perception of physical function at 1 year after TKA. Therefore, our results suggest that the intervention used in this study may be beneficial for early rehabilitation after TKA with effects at 1 year after surgery.

## Conflict of Interest

The authors declare no conflicts of interest.
